# Cardiovascular and Lung Involvement in Patients with Autoimmune Pancreatitis

**DOI:** 10.3390/jcm9020409

**Published:** 2020-02-03

**Authors:** Sara Nikolic, Katharina Brehmer, Nikola Panic, Roberto Valente, J.-Matthias Löhr, Miroslav Vujasinovic

**Affiliations:** 1 Department of Medicine, Huddinge, Karolinska Institute, 141 86 Stockholm, Sweden; sara.nikolic@ki.se; 2 Department of Radiology, Karolinska University Hospital, 141 86 Stockholm, Sweden; katharina.brehmer@sll.se; 3 Department of Clinical Science, Intervention, and Technology (CLINTEC), Karolinska Institute, 141 86 Stockholm, Sweden; matthias.lohr@ki.se; 4 Department for Digestive Diseases, Karolinska University Hospital, 141 86 Stockholm, Sweden; nikola.panicmail@gmail.com (N.P.); roberto.valente@sll.se (R.V.)

**Keywords:** autoimmune, pancreatitis, immunoglobulin G4, lung, cardiovascular

## Abstract

Introduction: Immunoglobulin G4-related disease (IgG4-RD) is a systemic immune-mediated disease characterised pathologically by the infiltration of IgG4-bearing plasma cells into the involved organs. Autoimmune pancreatitis (AIP) is a form of chronic pancreatitis with a heavy lymphocytic infiltration and two distinct histopathological subtypes, namely: lymphoplasmacytic sclerosing pancreatitis (AIP type 1) and idiopathic duct-centric pancreatitis (AIP type 2). Lung involvement and aortic involvement have been reported in 12% and 9% of patients with systemic IgG4-RD, respectively. In series including patients with AIP, both lung and aortic involvement were described in 2% of the patients. Most of the epidemiological data come from Japan, and there is a lack of information from Europe, especially the Scandinavian countries. Patients and methods: We performed a single-centre retrospective study on a prospectively collected cohort of patients diagnosed with AIP at the Department for Digestive Diseases at Karolinska University Hospital in Stockholm, Sweden, from 2004 to 2019. Demographic and clinical data were collected from the medical charts. Results: One hundred and thirty-three patients with AIP were analysed. Six patients were excluded because they lacked some of the clinical data relevant to the study. Demographic and clinical features of 127 patients were presented. There were 98 patients with AIP type 1-35 (35.7%) female and 63 (64.3%) male, with a mean age of 55.4 ± 18.2. Among them, 15 (15.3%) patients had lung and/or cardiovascular involvement-11 (11.2%) patients had lung involvement, 10 (10.2%) patients had cardiovascular involvement (six patients had both). Most of them (67.0%) had never smoked. The mean follow-up time of the patients with AIP type 1 was 49 months. Conclusions: Lung and/or cardiovascular involvement were diagnosed in 15 (15.3%) patients in our historical cohort of patients with AIP type 1. Most of the lung involvement was presented in the form of nodular lesions in the lungs, non-specific infiltrates, “ground-glass” appearance with pleura thickening, and effusion. Aortic involvement was a major form of vascular involvement in patients with AIP, as in previous published studies on patients with IgG4-RD.

## 1. Introduction

Immunoglobulin G4-related disease (IgG4-RD) is a systemic immune-mediated disease characterised pathologically by the infiltration of IgG4-bearing plasma cells into the involved organs [1]. Autoimmune pancreatitis (AIP) is a particular form of chronic pancreatitis with a heavy lymphocytic infiltration and two distinct histopathological subtypes, namely: lymphoplasmacytic sclerosing pancreatitis (LPSP; AIP type 1) and idiopathic duct-centric pancreatitis (IDCP; AIP type 2) [2].

AIP type 1 is considered to be a part of IgG4-RD, but the IgG4 values in the serum are not necessarily elevated in all of the patients. According to the International Consensus Diagnostic Criteria (ICDC), the diagnosis of AIP is based on the presence of one or more of the following factors: pancreatic parenchyma and pancreatic duct imaging, serum IgG4 level, other organ involvement (OOI), histology of the pancreas, and response to steroid treatment [3].

IgG4-RD is a relatively new clinical entity, and there is no specific definition of its epidemiology because of the multiple knowledge gaps and the fact that many physicians are unfamiliar with the diagnosis [4]. According to our recently published single-centre data, OOI was present in 84% of patients with AIP [5], which is higher when compared with other European studies, which vary from 47% to 61% [6,7,8,9].

Asthma and autoimmune diseases are a result of a dysregulated immune system [10], and pulmonary involvement (including lung, pleura, and mediastinum) has been reported in 12% of patients with systemic IgG4-RD presentations, and in 2% of patients in pancreatobiliary series [4]. Aortic involvement has been reported in 9% of patients with systemic IgG4-RD presentations and in 2% of patients in pancreatobiliary series [4]. Most of the epidemiological data comes from Japan, and there is a lack of information from Europe, especially the Scandinavian countries.

## 2. Patients and Methods

We performed a single-centre retrospective study on a prospectively collected cohort of patients with AIP diagnosed at the Department for Digestive Diseases at Karolinska University Hospital in Stockholm, Sweden, from 1 January 2004 to 1 December 2019.

Consecutive patients with a diagnosis of AIP according to the ICDC were included in the study. A retrospective analysis and diagnosis according to the ICDC criteria were performed for patients diagnosed in the period before the publication of ICDC [3]. The re-assessment of the ICDC classification was done by two independent senior investigators.

The following demographic and clinical data were collected from the medical records: gender, age, type of AIP (definite or probable-type 1, type 2, and non-otherwise specified (AIP NOS)), follow-up period (defined as the period between date of AIP diagnosis and the last contact with the patients), treatment of AIP, time of lung/cardiovascular involvement, type of lung/cardiovascular involvement (single nodular lesion, multiple nodular lesions, vasculitis, aortic involvement, cardiac involvement), duration of lung/cardiovascular involvement (defined as the period from the lung/cardiovascular disease diagnosis and last contact with the patient), treatment of lung/cardiovascular disease, other organ involvement (other than pancreas, lungs, heart, and vessels), smoking status (described as never, former, and active smokers), and clinical status at the last contact with patients regarding pancreas and lung disease (stable disease or relapse).

### 2.1. Ethics

The study was approved by the Clinic Ethical Committee in Stockholm (no. 2016/1571-31) and adheres to the latest version of the Declaration of Helsinki.

### 2.2. Statistics

A comparison of the data between the groups was evaluated using the t-test, chi-square, or Fisher’s test, where appropriate. A statistical analysis was performed using Stata software version 12 (Stata Corp, College Station, TX, USA).

## 3. Results

**Figure 1:**
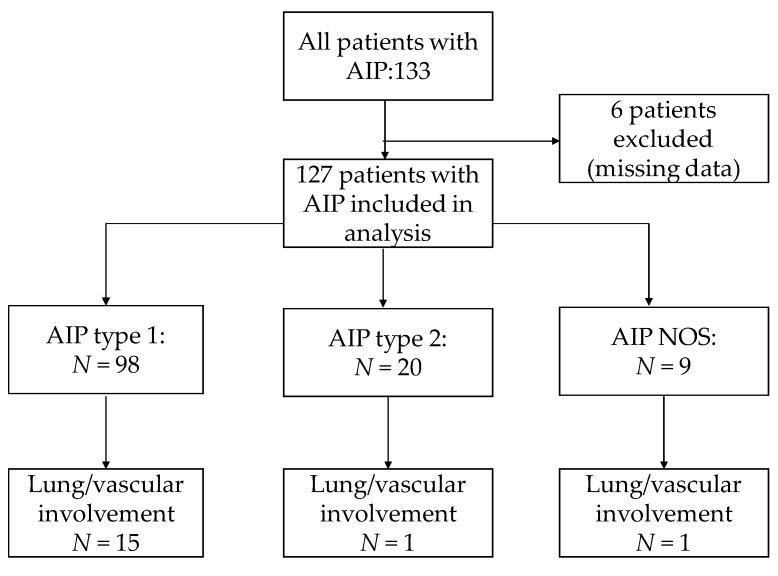
Flow chart of the included patients.

One hundred and thirty-three patients with AIP were analysed (Figure 1 and Table 1). Six patients were excluded, as they lacked some of the clinical data relevant to the study. The demographic and clinical features of 127 patients are presented in Table 1.

There were 98 patients with AIP type 1-35 (35.7%) female and 63 (64.3%) male, and the mean age was 55.4 ± 18.2. Among them, 15 (15.3%) patients had lung and/or cardiovascular involvement—11 (11.2%) patients with lung involvement, 10 (10.2%) patients with cardiovascular involvement (6 patients had both). Most of them (67.0%) had never smoked. The mean follow-up period of the patients with AIP type 1 was 49 months.

**Figure 2:**
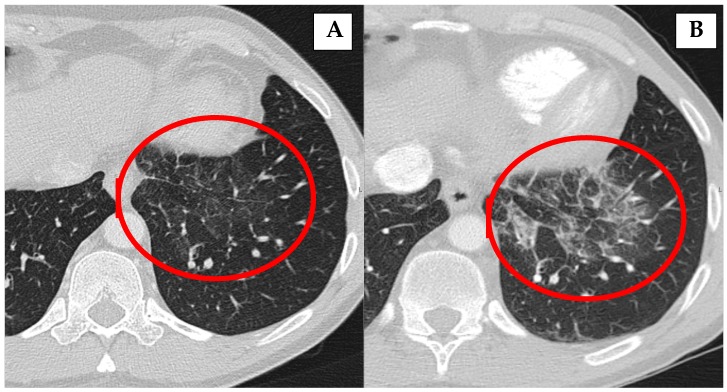
Inflammatory changes in the lungs (**B**) that decrease in size and disappear after corticosteroid treatment (**A**).

Most of the lung involvement in the patients with AIP type 1 (Table 2) was presented in the form of nodular lesions in lungs (54.4%), non-specific infiltrates (36.4%), “ground-glass” appearance (18.2%), pleura thickening (18.2%), pleural effusion (18.2%), and mediastinal lymph nodes enlargement (9.1%). In 36.4% of the patients, more than one form of lung involvement was presented. In seven patients (63.6%), lung involvement occurred after the diagnosis of AIP type 1; in three patients (27.3%), at the time of AIP diagnosis, and in one (9.1%) patient, before the diagnosis of AIP. One (9.1%) patient had a relapse of lung involvement (Figure 2).

Asymmetric thickening of aorta wall was the most common (60%) form of vascular involvement (Table 3). Granulomatosis with polyangiitis (GPA) was present in two (20%) patients, Churg Strauss in one (10%) patient, and vasculitis in the form of skin changes in one (10%) patient. Cardiac involvement was seen in two (20%) patients in the form of eosinophilic myocarditis.

The distribution of the demographic and clinical features of the patients included in relation to the presence of lung/cardiovascular involvement showed no significant difference regarding AIP type, gender, age, treatment, or smoking (Table 1). OOI (Sjögren disease, sialadenitis, dacryoadenitis, and kidney involvement) was more common in patients with lung/cardiovascular involvement.

## 4. Discussion

Lung and/or cardiovascular involvement were diagnosed in 15 (15.3%) out of 98 patients with AIP type 1.

Most of the lung involvement presented in the form of nodular lesions in the lungs, non-specific infiltrates, “ground-glass” appearance with pleura thickening, and effusion (Table 2). The pulmonary manifestation of IgG4-RD according to anatomic compartments can generally be categorised as parenchyma (nodules, masses, and interstitial lung disease), airway (stenosis, endobronchial mass, and bronchospastic disease), vasculature (vasculitis and pulmonary hypertension), mediastinum (lymphadenopathy and fibrosing mediastinitis), and pleural (thickening mass, and effusion) involvement [11].

In a cross-sectional study of 114 patients, thoracic involvement (lung and pleura) was found in 16 (14%) patients [12]. Zen and colleagues reported 21 patients with IgG4-related lung and pleural involvement, and of these patients, 47% had subjective pulmonary symptoms (the others were asymptomatic and diagnosed on routine work-up) [13]. In another report from the same group, nine cases of inflammatory pseudotumor of the lungs were reported (two out of nine were symptomatic) [14]. Taniguchi and colleagues described a case of interstitial pneumonia associated with AIP [15], and Yamashita and colleagues reported three cases of interstitial lung disease with a common histology and abundant IgG4-positive cell infiltration [16]. Matsui and colleagues reported 18 patients with lymphadenopathy, thickening of the bronchial wall, nodules, subpleural, and peribronchovascular consolidation [17].

The aortic involvement in our group of patients was a major form of vascular involvement in the patients with AIP, as in previous published studies on patients with IgG4-RD [4]. Other vascular involvement has been rarely reported [4,18]. In our patients, no aortic dilation or aneurysms were diagnosed.

Granulomatosis with polyangiitis (GPA), formerly known as Wegener’s granulomatosis, and Churg-Strauss was diagnosed in four patients and successfully treated with rituximab (Table 2 and Table 3).

In a recently published retrospective analysis, Yoo and colleagues investigated whether elevated serum IgG4 at the time of diagnosis of microscopic polyangiitis (MPA) and GPA may be associated with concurrent IgG4-related disease (IgG4-RD) in immunosuppressive drug-naïve patients [19]. The mean serum IgG did not vary between the groups, and among the 46 patients, they could not find patients who could be classified as IgG4-RD. Although GPA is not part of IgG4-RD, it is worth mentioning that in the context of OOI, it is an important differential diagnosis (which is why it was included in our analysis). It is important to exclude other diseases from the same group (diseases with multisystem involvement and diseases with pulmonary inflammation and increased IgG4 plasma cells), such as connective tissue diseases with lung involvement, sarcoidosis, eosinophilic granulomatous with polyangiitis (Churg-Strauss), multicentric Castelman’s disease, and Rosai–Dorfman disease [11]. The results of the most relevant studies are presented in Table 4 and Table 5.

In 18 out of 127 patients (14.2%) with AIP, we diagnosed asthma alone or in combination with other clinical conditions (Table 1). Asthma is traditionally considered as being a heterogeneous respiratory condition clinically characterised by airway inflammation, reversible airflow obstruction, and airway hyperresponsiveness, but the recent discovery of novel pathogenic effector cells has led to theories of common underlying pathophysiological pathways [10]. Corticosteroids (CST) are the cornerstone of the treatment of both AIP and the inflammatory component of asthma, just like all other autoimmune diseases. The presence of autoantibodies in the lungs could be one of the mechanisms that contribute to the observed steroid sub-sensitivity in severe asthmatics [10,25]. Ito and colleagues reported three cases of bronchial asthma preceding IgG4-related autoimmune pancreatitis by three months to 30 years [26].

In a nationwide study conducted in Sweden, standardised incidence ratios (SIRs) were calculated for the subsequent autoimmune diseases in 4006 patients who were hospitalised for an autoimmune condition after the last hospitalisation for asthma [27]. The SIRs were increased for 11 subsequent autoimmune conditions diagnosed at least five years following asthma diagnosis. As a result of the relatively low number of included patients and limited data, it is not possible to draw any strong conclusions regarding a possible association between asthma and AIP. However, the presence of asthma in 14.2% of our patients could be a good starting point for further studies on this topic. Of course, it is not possible to exclude the possible interaction with environmental factors.

De Buy Wenniger and colleagues hypothesised that professions traditionally considered as “blue collar”, as well as chronic occupational antigen exposure, could play a crucial pathogenic role for the mainly elderly male IgG4-RD patients in a Dutch cohort of 25 patients (IgG4-associated cholangitis and AIP) [28]. However, an analysis of the job history of our group of patients could not confirm the Dutch hypothesis, as only 29.5% of patients belonged to “blue collar” professions, and there was no significant difference between patients with and without lung/cardiovascular involvement.

Another interesting finding was the fact that lung/cardiovascular involvement was presented not only in patients with AIP type 1 (which is considered to be part of IgG-RD), but also in patients with AIP type 2 (one patient) and AIP-NOS (one patient), which are not associated with IgG4-RD.

**Figure 3:**
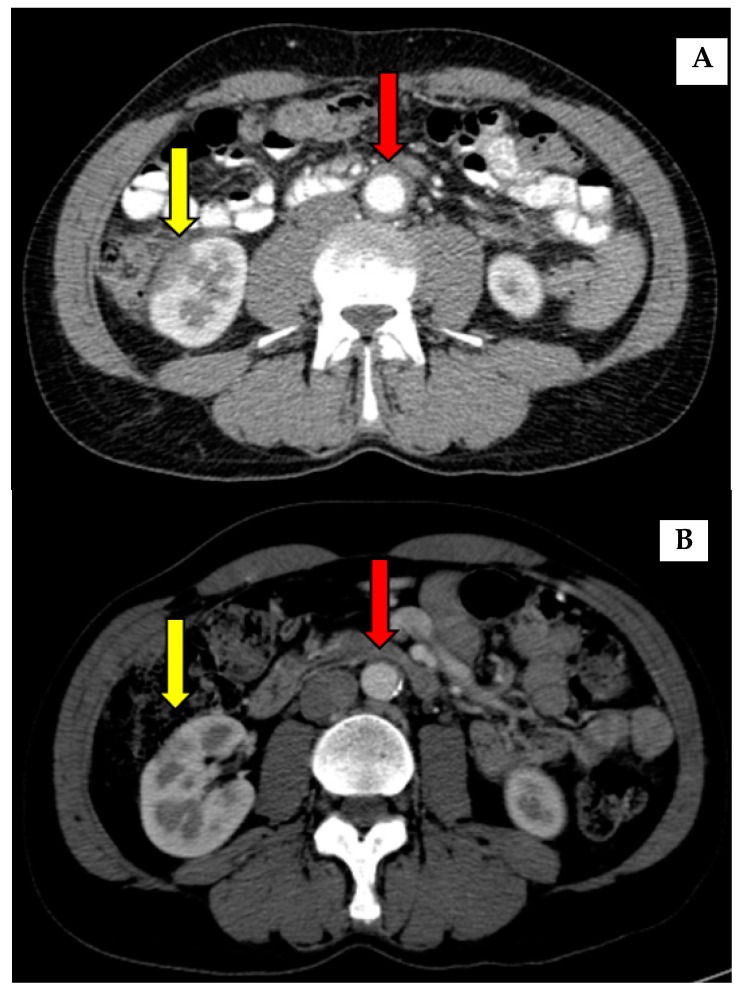
Inflammatory changes in aorta-thickening of aorta wall before (**A**) and after (**B**) corticosteroid treatment (red arrow). An improvement of kidney involvement after the treatment was also seen (yellow arrow).

In most of our patients, lung involvement and vascular involvement were diagnosed with imaging as part of the AIP work-up, and none of the patients with nodular lesions were symptomatic (except for patients with GPA and Churg-Strauss syndrome who were treated via rheumatologists). Patients with AIP responded excellently to initial CST (Figure 2 and Figure 3), but relapses of the disease are common and other types of treatment are necessary (Table 1).

Heterogeneous groups of patients with lung/cardiovascular involvement present a weakness in the presented study. The authors are aware that GPA and asthma cannot be considered as being a direct part of IgG4-RD, but the purpose of the study was to present all patients with all kinds of lung and cardiovascular involvement, as this is extremely important from a clinical point of view, especially for gastroenterologists and surgeons, who meet these patients and must be aware of possible other organ involvement and its clinical importance.

It is still the case that most of the data on this topic come from Japan, despite the fact that IgG4-RD has been described in nearly all racial and ethnic groups [4]. Further studies on different aspects of IgG-RD and AIP should be strongly encouraged in order to increase the awareness of the disease among physicians, and from this point of view, our results show the significance of the study and that it constitutes an important contribution to an improved understanding of the natural course of AIP.

## 5. Conclusions

Lung and/or cardiovascular involvement was diagnosed in 15 (15.3%) patients in our historical cohort of patients with AIP type 1. Most of the lung involvement was presented in the form of nodular lesions in the lungs, non-specific infiltrates, “ground-glass” appearance with pleura thickening, and effusion. Aortic involvement was a major form of vascular involvement in patients in AIP, as in previous published studies on patients with IgG4-RD. From a clinical point of view, increasing awareness of AIP and other organ involvement is necessary for all types of clinicians.

## Figures and Tables

**Table 1:** Demographic and clinical characteristics of patients.

Parameter	AIP with Vascular/Lung Involvement	AIP without Vascular/Lung Involvement	*p*
Number (%)	17 (13.4%)	110 (86.1%)	
AIP type			0.457
Type 1	15 (88.2%)	83 (75.4%)	
Type 2	1 (5.9%)	19 (17.3%)	
Not otherwise specified	1 (5.9%)	8 (7.3%)	
Gender			0.960
Female	7 (41.2%)	46 (41.8%)	
Male	10 (58.8%)	64 (58.2%)	
Age *	59.76 ± 16.36	51.00 ± 19.58	0.083
Follow-up ** (months)	60.43 ± 52.60	45.88 ± 44.51	0.223

AIP-autoimmune pancreatitis; AIP-NOS-autoimmune pancreatitis not otherwise specified; * age at the time of AIP dg; ** period between AIP dg and time of last contact with patient;

**Table 2:** Demographic and clinical features of patients with AIP type 1 and lung involvement.

*N*	Gender	Age	Type of AIP	Onset and Type of Lung Involvement	Treatment	Smoking Status
1	female	53	Type 1	1 year after AIP diagnosis: “ground-glass” appearance; relapse after 12 years of AIP diagnosis with pleura thickening and mediastinal lymph nodes enlargement	No treatment	Former (13 PY)
2 *	male	66	Type 1	4 years after AIP diagnosis: non-specific infiltrates in both lung lobes	Azathioprine and CST 10 mg	Never
3 *	male	66	Type 1	9 months after AIP diagnosis: nodular lesions in lungs	Rituximab and CST 2.5 mg	Never
4 *	female	65	Type 1	2 months after AIP diagnosis: nodular lesions in lungs	Rituximab	Never
5 *	male	85	Type 1	At the time of AIP diagnosis: non-specific infiltrates in both lung lobes	Previously treated with CST, currently no treatment	Former (10 PY)
6 *	female	24	Type 1	At the time of AIP diagnosis: infiltrates in both lung lobes with pleural effusion	Previously treated with CST, currently no treatment	Never
7 *	male	50	Type 1	5 years before AIP diagnosis: Churg Strauss syndrome, lung infiltrates, patients also had Erdheim Chester disease and hyper eosinophilic syndrome	Rituximab	Never
8	female	73	Type 1	5 months after AIP diagnosis: nodular lesions in lungs	Previously treated with CST, currently no treatment	Never
9	female	39	Type 1	At the time of AIP diagnosis: lesions in lungs and pleural effusion	No treatment	Former (2 PY)
10	female	60	Type 1	3 years after AIP diagnosis: “ground-glass” appearance and pleura thickening	Previously treated with CST, currently no treatment	Former (10 PY)
11	male	69	Type 1	5 years after AIP diagnosis: nodular lesions in lungs	Previously treated with CST, currently no treatment	Never

AIP-autoimmune pancreatitis; * patients also in Table 3; PY-pack-years of smoking; CST-corticosteroids; age-age of patients at the time of AIP diagnosis.

**Table 3:** Demographic and clinical features of patients with AIP type 1 and cardiovascular involvement.

*N*	Gender	Age	Type of AIP	Onset and Type of Vasculitis	Treatment	Smoking Status
1	male	75	Type 1	4 years after AIP diagnosis: asymmetric thickening of aorta wall (up till 5 mm) in infrarenal part of aorta	Rituximab and CST 10 mg	Former (30 PY)
2 *	male	66	Type 1	4 years after AIP diagnosis: thickening of aorta wall over the bifurcation	Azathioprine and CST 10 mg	Never
3	male	57	Type 1	At the time of AIP diagnosis: circumferential thickening of aorta wall	Rituximab	Never
4 *	male	66	Type 1	GPA 9 months after AIP: diagnosis with lung and bowel involvement	Rituximab and CST 2.5 mg	Never
5 *	female	65	Type 1	GPA 2 months after AIP: diagnosis with lung involvement	Rituximab	Never
6	male	65	Type 1	1 year after AIP diagnosis: mild thickening in infrarenal part of aorta	No treatment so far	Never
7	male	68	Type 1	3 months after AIP diagnosis: vasculitis in form of skin changes	Previously treated with CST and now hematologic treatment with lenalidomide (multiple myeloma)	Former (8 PY)
8 *	male	85	Type 1	At the time of AIP diagnosis: imaging signs of periaortitis	Previously treated with CST, currently no treatment	Former (10 PY)
9 *	female	24	Type 1	At the time of AIP diagnosis: eosinophilic myocarditis	Previously with CST, currently no treatment	Never
10 *	male	50	Type 1	5 years before AIP diagnosis: Churg Strauss syndrome, pericarditis and eosinophilic myocarditis, patients also had Erdheim Chester disease and hyper eosinophilic syndrome	Rituximab	Never

AIP-autoimmune pancreatitis; CST-corticosteroids; PY-pack-year; * patients also in Table 2; GPA-granulomatosis with polyangiitis (formerly known as Wegener’s granulomatosis); AIP NOS-autoimmune pancreatitis non-otherwise specified; age-age of patients at the time of AIP diagnosis.

**Table 4:** Studies on vascular involvement in patients with IgG4-related diseases (IgG4-RD).

Author	Year	Country	Patients	Age/ Gender	Vascular Involvement
Ozawa [20]	2017	Japan	179 patients with IgG4-RD	67 years/ 73.2% male	Periaortitis/periarteritis: 36.3%
Perugino [21]	2015	USA	160 patients with IgG4-RD	54.6 years/ 78% male	Large-vessel involvement: 22.5%
Yabusaki [22]	2017	Japan	37 patients with IgG4-RD	68 years/ 64.9% male	Aortitis: 41%
Inoue [23]	2015	Japan (8 centres)	235 patients with IgG4-RD	67 years/ 80.4% male	Aorta involvement: 8.5%
Brito-Zeron [4]	2014	Review (North America, Europe and Asia)	3482 reported cases of IgG4-RD	Not reported	Aortic involvement: 35/375 (9%) in systemic series 5/313 (2%) in pancreatobiliary series 36/268 (13%) in other series
Presenting study	2019	Sweden	98 patients with autoimmune pancreatitis type 1	55.4 years/ 60.9% male	10.2%

**Table 5:** Studies on lung involvement in patients with IgG4-related diseases (IgG4-RD).

Author	Year	Country	Patients	Age/ Gender	Lung Involvement
Wallace	2015	USA	125 patients with IgG4-RD	50.3 years/ 61% male	17.6%
Zen [12]	2010	Japan	114 patients with IgG4-RD	65 years/ 76.3% male	9.6%
Brito-Zeron [4]	2014	Review (North America, Europe and Asia)	3482 reported cases of IgG4-RD	Not reported	75/620 (12%) in systemic series 18/237 (8%) in glandular series 6/313 (2%) in pancreatobiliary series 75/253 (30%) in other series
Fernandez-Codina [24]	2015	Spain (14 centres)	55 patients with IgG4-RD	53 years/ 69.1% male	9%
Inoue [23]	2015	Japan (8 centres)	235 patients with IgG4-RD	67 years/ 80.4% male	5.5%
Ogoshi	2015	Japan	35 patients with autoimmune pancreatitis	67 years/ 68.6% male	40%
Presenting study	2019	Sweden	98 patients with autoimmune pancreatitis type 1	55.4 years/ 60.9% male	11.2%
